# Association of prenatal parabens exposure with adverse pregnancy outcomes: the role of prenatal depressive symptoms

**DOI:** 10.3389/fpubh.2026.1802833

**Published:** 2026-04-23

**Authors:** Mengjun Chang, Xianjia Li, Ye Li, Yueyi Lv, Jin Ji, Can Liu, Yunyun Du, Shuqin Ma, Suzhen Guan

**Affiliations:** 1 School of Public Health, Ningxia Medical University, Yinchuan, China; 2 Key Laboratory of Environmental Factors and Chronic Disease Control, Yinchuan, China; 3 General Hospital of Ningxia Medical University, Yinchuan, China

**Keywords:** adverse pregnancy outcomes, effect modification, mixture exposure, parabens, prenatal depressive symptoms

## Abstract

**Background:**

Parabens (PBs) are common endocrine-disrupting preservatives linked to adverse pregnancy outcomes, while prenatal depressive symptoms are also prevalent and harmful. Their combined effects remain poorly understood.

**Objective:**

To examine individual and joint associations of prenatal PBs exposure with adverse pregnancy outcomes (APOs) and to assess modification by maternal depressive symptoms.

**Methods:**

A prospective cohort of 934 mother-infant pairs from Ningxia, China, was analyzed. Maternal serum concentrations of five PBs were measured by HPLC-MS/MS. Depressive symptoms were assessed using the Edinburgh Postnatal Depression Scale. APOs included small vulnerable newborns, macrosomia, fetal distress, and asphyxia. Logistic regression and quantile g-computation were used, with stratified and interaction analyses.

**Results:**

In the overall cohort, propylparaben (PrP) was significantly associated with fetal distress (OR = 1.25, 95% CI: 1.05–1.49), while mixed PBs exposure increased the risk of macrosomia (OR = 1.62, 95% CI: 1.06–2.48). Quantile g-computation revealed that ethylparaben (EtP), PrP, and heptylparaben (HeP) were the primary contributors to the mixture effect on macrosomia. Maternal depressive symptoms significantly modified these associations: among women with depressive symptoms (*n* = 418), PrP was associated with overall APOs (OR = 1.21, 95% CI: 1.03–1.41), and EtP was linked to small vulnerable newborns (OR = 1.20, 95% CI: 1.04–1.38). In contrast, no significant associations were observed in women without significant depressive symptoms. Interaction analyses confirmed significant effect modification by depressive symptoms for PrP (*P*-int = 0.049) and EtP (*P*-int = 0.024).

**Conclusion:**

Prenatal PBs exposure is associated with increased APOs risk, particularly among women with depressive symptoms, underscoring the importance of integrating environmental and mental health considerations into prenatal care.

## Graphical abstract

**Figure:**
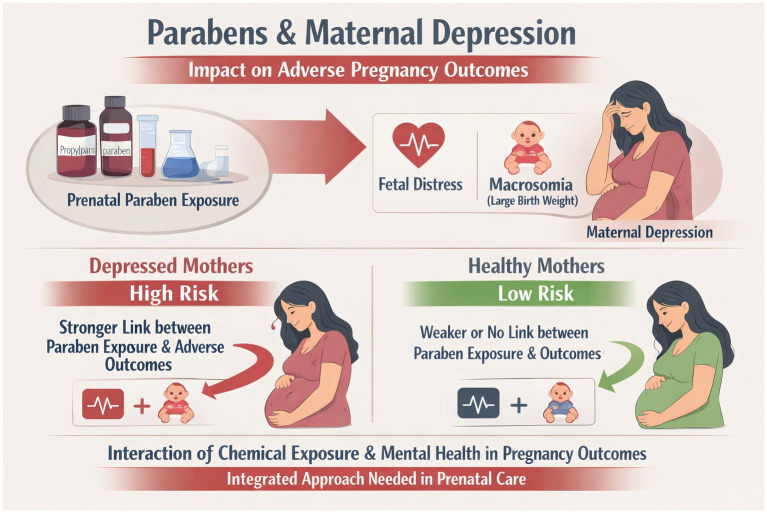

## 1 Introduction

Parabens (PBs) are widely used preservatives with antimicrobial properties, commonly present in personal care products, pharmaceuticals, foods, and industrial materials. Due to their strong antimicrobial activity, low cost, and low sensitization potential, parabens are widely used. Structurally, PBs are esters of para-hydroxybenzoic acid, among which methylparaben and propylparaben are the most commonly used. However, because of their estrogenic potential, they are widely recognized as potential endocrine-disrupting chemicals (EDCs) and are subject to regulatory restrictions in regions such as the European Union, the United States, and Canada (1). Parabens may interfere with natural development by mimicking or blocking natural sex hormones, leading to phenotypic variations, birth defects, and recurrent miscarriages. The prenatal stage of human development is one of the most active periods of life, during which hormonal signaling plays a crucial role in fetal development (2–5). Recent studies have found that parabens may have adverse effects on fetuses and newborns, with a large prospective analysis of multiple U. S. birth cohorts reporting associations with reduced birth weight and increased risk of small-for-gestational-age outcomes (6–8). Growing evidence suggests that parabens can cross the placental barrier and have been detected in multiple maternal–fetal compartments, including maternal serum, cord blood, and amniotic fluid, indicating potential fetal exposure during pregnancy (9, 10). These findings highlight the potential risks of prenatal paraben exposure, particularly during critical windows of fetal development. Therefore, further research on the effects of parabens on fetuses and newborns, as well as their potential mechanisms of transfer from mother to fetus, is crucial for assessing their long-term impact on human health.

The prevalence of depression and anxiety during pregnancy, and their effects on pregnancy outcomes, are significant areas of ongoing research. Studies have shown that the prevalence of depression during pregnancy varies depending on the diagnostic criteria used, with the highest rate reaching up to 16%, of which about 5% are severe cases (11). Although there is a lack of a unified screening tool, research has shown that a considerable number of pregnant women experience anxiety/depression during pregnancy, including worries about pregnancy itself (12, 13). In addition, there is substantial evidence of exposure to stress during pregnancy, especially in certain specific populations. For example, a study of a diverse urban sample found that 78% of pregnant women experienced low to moderate levels of prenatal psychosocial stress, while 6% experienced high levels of stress (14). Globally, common stressors for pregnant women include lack of economic resources, unfavorable employment conditions, heavy family and household responsibilities, intimate relationship tension, and pregnancy complications. An increasing number of studies have shown that stress and emotional states during pregnancy, especially depression and anxiety, are closely related to adverse birth outcomes such as preterm birth and low birth weight (15, 16). It is estimated that about two-thirds of low birth weight infants are preterm, reflecting that preterm birth is a direct cause of low birth weight due to reduced intrauterine growth time (17, 18). Therefore, in-depth research on the impact of prenatal depressive symptoms on pregnancy outcomes is of great significance for improving maternal and child health.

Although there have been numerous studies that have separately investigated the potential impacts of parabens exposure and prenatal psychological depression on pregnancy outcomes, research on the interactive effects of these two factors remains relatively limited. Parabens, as endocrine disruptors, may interfere with fetal development by disrupting hormone signaling pathways. Meanwhile, psychological states such as prenatal depressive and anxiety symptoms can also affect hormone levels, thereby influencing pregnancy outcomes. Therefore, the interaction between paraben exposure and prenatal depressive symptoms may have a synergistic impact on adverse pregnancy outcomes. Given the widespread use of parabens and the high prevalence of prenatal depressive symptoms, a thorough investigation into their combined effects on adverse pregnancy outcomes, such as preterm birth and low birth weight, is essential. This research not only aids in assessing the potential health risks of parabens more comprehensively, but also provides a scientific basis for developing more effective preventive strategies and interventions, ultimately better protecting maternal and infant health.

## 2 Materials and methods

### 2.1 Study design and participants

Between June 2023 and March 2025, 1,667 pregnant women in their mid-pregnancy undergoing routine antenatal checkups were recruited from a tertiary hospital in Ningxia Hui Autonomous Region. After excluding multiple pregnancies, pregnancy loss (miscarriage or stillbirth), and cases lacking birth-outcome information, 934 singleton live-born pregnancies were available for analysis. All participants met the following criteria: maternal PBs measured during pregnancy, at least one mid-pregnancy psychological-stress assessment completed, and complete birth-outcome data for the offspring. All subjects signed an informed consent form prior to data collection (see Supplementary Figure 1). Ethical approval was obtained from the Ethics Committee of Ningxia Medical University before the start of the study (approval number: Ningxia Medical University Ethics No. 2022·007). Trial registration: Not applicable.

### 2.2 Diagnosis of adverse pregnancy outcomes

Pregnant women who met any of the following criteria were classified as having adverse pregnancy outcomes (APOs): (1) Small vulnerable newborn (SVN): defined according to Ashorn et al. (19) as any liveborn infant who is either preterm (born before 37 completed weeks of gestation), small for gestational age (SGA, birthweight below the 10th centile of the sex-specific birthweight for gestational age standard), or has low birthweight (LBW, <2,500 g). (2) Macrosomia: defined as birthweight ≥4,000 g. (3) Fetal distress: diagnosed based on abnormal fetal heart rate patterns, meconium-stained amniotic fluid, or abnormal umbilical artery pH (<7.20) during labor. (4) Asphyxia: defined as an Apgar score <7 at 5 min after birth.

### 2.3 Measurement of PBs levels in mothers

In the present study, peripheral blood samples were collected from pregnant women at the time of enrollment. The samples were immediately centrifuged at 3000 rpm for 15 min to isolate the serum, which was stored at −80 °C to avoid degradation. The levels of PBs in maternal serum were determined by high performance liquid chromatography–tandem mass spectrometry (HPLC-MS/MS). A total of five PBs congeners were analyzed, including methylparaben (MeP), ethylparaben (EtP), propylparaben (PrP), butylparaben (BuP), and heptylparaben (HeP). The intra-and inter-batch coefficients of variation were less than 10% for all PBs congeners, and the limits of detection (LODs) ranged from 0.07 to 0.4 ng/mL. Concentrations below the LOD were imputed using a value of LOD/ $\sqrt{2}$ .

### 2.4 Assessment of prenatal depressive symptoms

To assess psychological depression during pregnancy, the Edinburgh Postnatal Depression Scale (EPDS) was used. The EPDS was developed by Cox et al. in 1987 and is a self-report questionnaire widely used for the initial screening of postpartum depression (20). The scale began to be widely used in China for postpartum depression screening in the 1990s. Numerous studies have demonstrated that the EPDS is effective for screening both postpartum depression and depression during pregnancy. The EPDS consists of 10 items, which can be further divided into three subscales: emotional (items 1–2), anxiety (items 3–6), and depression (items 7–10). Each item is rated on a 4-point scale, with scores ranging from 0 to 3 based on symptom severity: 0 (never), 1 (occasionally), 2 (often), and 3 (always). The maximum score for the scale is 30 points, with higher scores indicating more depressive symptoms. The Chinese version of the EPDS was translated and adapted by Lee et al. from the Chinese University of Hong Kong in 1998, with a sensitivity of 82% and specificity of 86% for the Chinese version (21). The Chinese version of the EPDS was validated in the Chinese population, with a cutoff of ≥9 for screening depressive symptoms (Cronbach’s *α* = 0.89) (22). In the present study, participants with an EPDS score ≥9 were classified as having significant depressive symptoms.

### 2.5 Covariates

In this study, we screened potential covariates using the LASSO method. The following covariates were included in the final analysis: Age (years), pre-pregnancy BMI (kg/m^2^), EPDS score, and number of pregnancies were treated as continuous variables. Place of residence was categorized as urban or rural. Personality was classified into three categories: introverted, ambiverted, and extroverted. Per capita monthly income was categorized as <2000 RMB, 2000–5,000 RMB, or >5,000 RMB. Mode of conception was categorized as natural conception or assisted reproductive technology. Intended pregnancy was categorized as planned pregnancy, natural conception, or unplanned pregnancy. Presence of underlying diseases was defined as having any of the following pre-existing conditions: mental disorders, diabetes, hypertension, or thyroid disease. Detailed distributions of these covariates are presented in Supplementary Table S1.

### 2.6 Statistical analysis

The data organization and statistical analysis of this study were done using SPSS software and R software. Before data analysis, all quantitative data were tested for normality, and the results showed that none of the data conformed to normal distribution. For continuous data with skewed distribution, they were described by median (M) and interquartile spacing (Q1, Q3), and the Mann–Whitney U test was used for between-group comparisons; categorical data were expressed as frequency (n) and percentage (%), and analyzed by the chi-square test or Fisher exact probability method, and the differences between the groups were considered to be statistically significant when the *p*-value was less than 0.05.

To address the skewed distribution of PBs exposure levels, natural logarithmic transformation of PBs concentrations was performed to satisfy the linearity assumption for subsequent statistical modeling. Prenatal depressive symptoms were assessed using the EPDS, and participants were categorized into two groups based on the established cutoff: the depressive symptoms group (EPDS ≥ 9) and the non-significant depressive symptoms group (EPDS < 9). To compare demographic characteristics and PBs levels between these two groups, t-tests or Wilcoxon rank-sum tests were used for continuous variables, and chi-square tests were used for categorical variables.

Binary logistic regression was used to examine the effect of PBs exposure on adverse pregnancy outcomes. The association between maternal depressive symptoms and adverse pregnancy outcomes was also assessed. Additionally, the quantile G-score (qgcomp) method was applied to further explore the combined effects of multiple PBs compounds on adverse pregnancy outcomes. All models were adjusted for age, pre-pregnancy BMI, EPDS score, place of residence, personality, per capita monthly income, number of pregnancies, mode of conception, intended pregnancy, and presence of underlying diseases. The odds ratio (OR) values and their 95% confidence intervals (CI) associated with each logarithmic increase in PBs concentration and adverse pregnancy outcomes were calculated.

To assess the potential moderating effect of maternal depressive symptoms, we first conducted stratified analyses based on EPDS scores (depressive symptoms group vs. non-significant depressive symptoms group). This allowed us to examine whether the association between PBs exposure and adverse pregnancy outcomes differed by maternal psychological status. Additionally, the interaction term between PBs exposure and maternal depressive symptoms was added to the model, and its significance was tested using the multivariate Wald test. A *p*-value <0.1 for the interaction was considered indicative of an interaction effect.

To validate the robustness of the results, we also conducted a sensitivity analysis to assess the stability and consistency of the results. The study excluded pregnant women with more than one miscarriage and reapplied the binary logistic regression model to the screened subsample to assess the association between individual PBs and adverse pregnancy outcomes. Additionally, the quantile qgcomp method was used to explore the combined exposure effects of multiple PBs. Additionally, we repeated the stratified analysis of depressive symptom status in this subsample and tested the interaction term between PBs and depressive symptom status. The sensitivity analysis maintained the same covariate-adjusted model as the main analysis.

## 3 Results

### 3.1 Characteristics of participants

**Figure 1:**
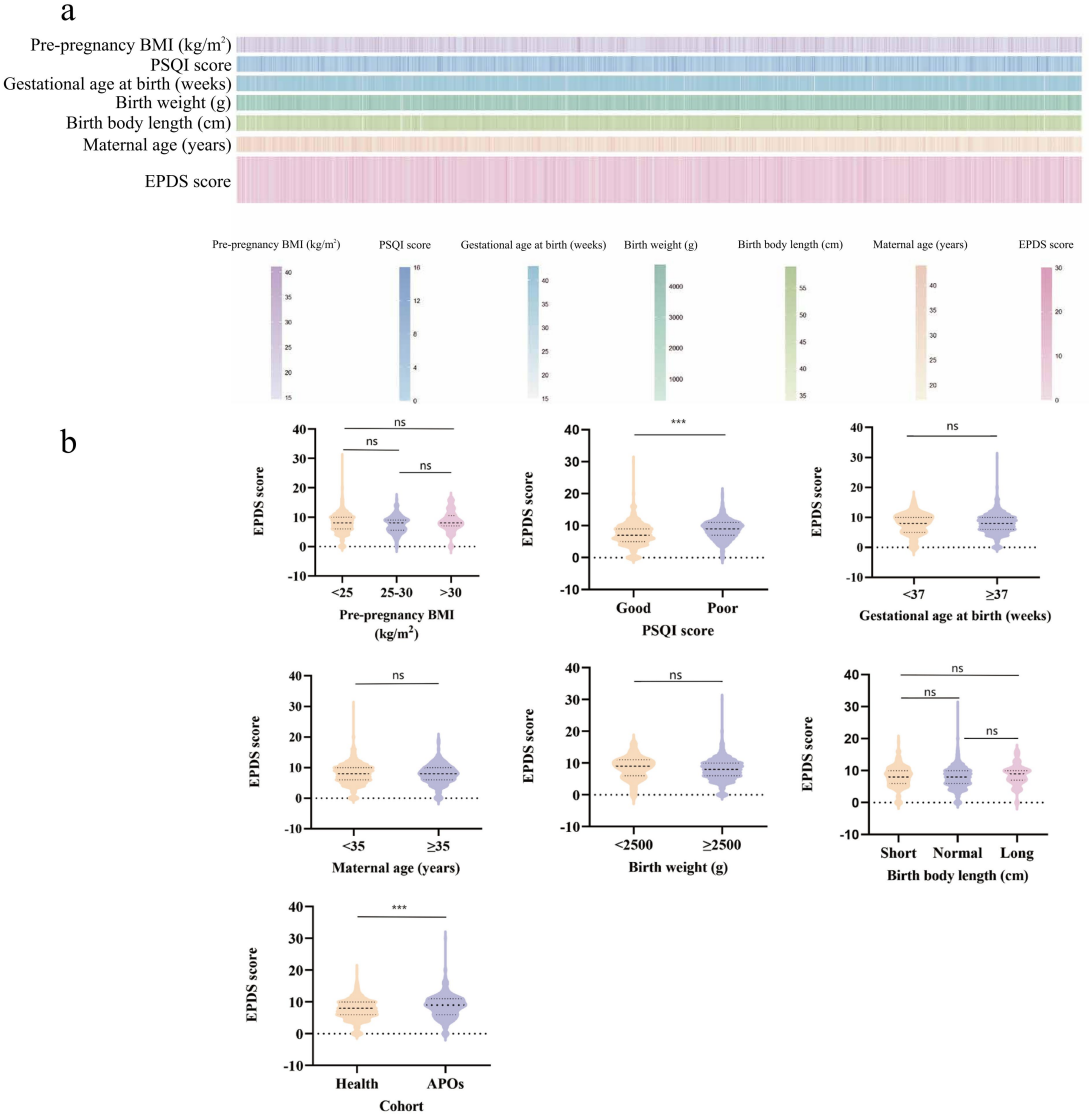
Distribution of maternal health indicators and correlation analysis with postpartum depression risk. **(a)** Distribution profiles of maternal health indicators. **(b)** Association between demographic variables and EPDS scores. ^*^p < 0.05, ^***^p < 0.001; ns non-significant.

As shown in Supplementary Table S1, the sample comprised 934 pregnant women aged 17 to 45 years. Based on the EPDS scores, 418 (44.8%) pregnant women were classified into the depressive symptoms group (EPDS ≥ 9), and the remaining 516 (55.2%) comprised the non-significant depressive symptoms group (EPDS < 9). Among them, 18.0% were over 35 years old, 19.5% had abnormal pre-pregnancy BMI, 76.3% had an educational background of college diploma or above, 85.6% were not only children, 11.1% had underlying diseases, and 40.9% had poor sleep quality. As shown in Figures 1a,b, the median EPDS score was significantly higher in pregnant women with poor sleep quality than in those with good sleep quality, based on the analysis grouped by PSQI scores. Additionally, we compared the EPDS scores between healthy pregnant women and those who experienced APOs. The results showed that the median EPDS score was significantly higher in the APOs group than in the healthy group (*p* < 0.05). As shown in Supplementary Table S2, regarding the basic information of the infants, there were 500 boys and 434 girls; 7.5% were low-birth-weight infants, and 24.2% had short body length. The findings indicated significant differences in birth weight and adverse pregnancy outcomes between infants of mothers in the depressive symptoms group and those in the non-significant depressive symptoms group (*p* < 0.05), while no significant differences were observed in other indicators (*p* > 0.05).

### 3.2 Covariate selection via LASSO

**Figure 2:**
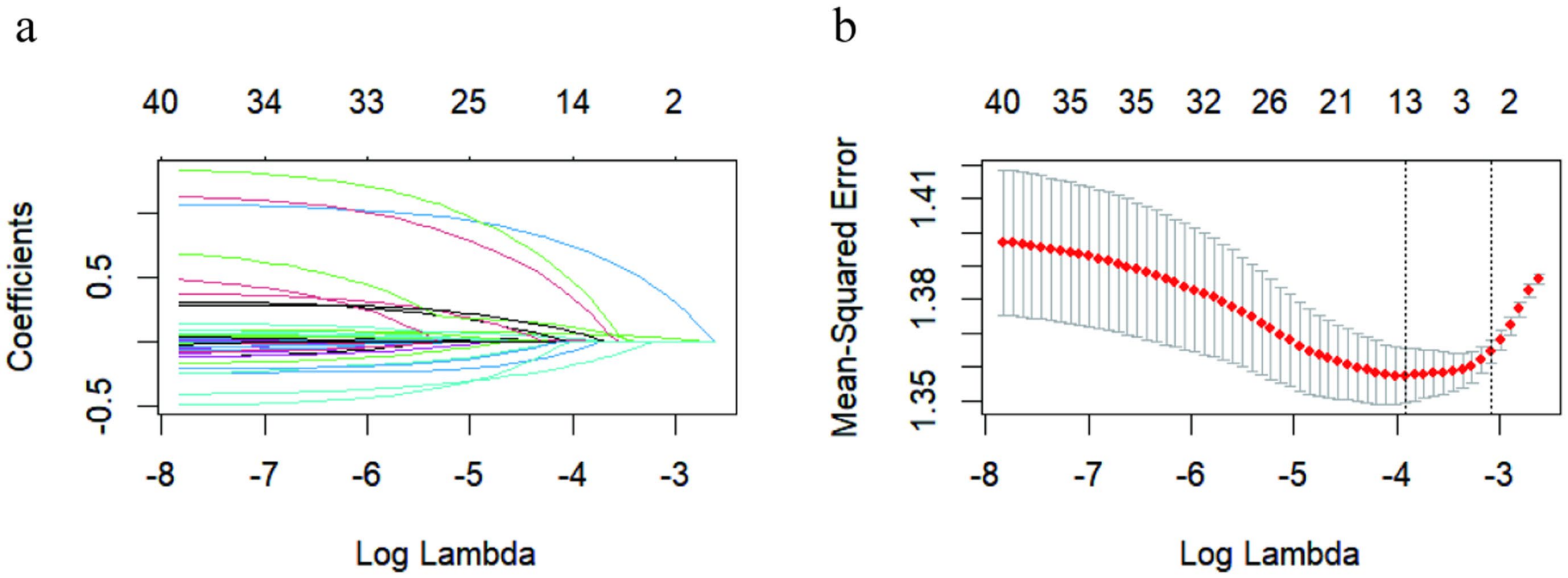
Predictors were chosen through LASSO regression. **(a)** LASSO coefficient curves were plotted for 40 variables in accordance with the log (lambda) series. **(b)** The LASSO regression employed 10-fold cross-validation. Binomial deviance was plotted against log (lambda). Vertical dashed lines were drawn at the optimal values based on the minimum value criterion (left dashed line) and the 1 standard error criterion (right dashed line).

A total of 40 potential risk variables were included in the LASSO analysis, as shown in Figures 2a,b. LASSO regression with 10-fold cross validation was performed to select the optimal penalty parameter lambda. The lambda that minimized the cross-validated deviance was used for variable selection. Under this criterion, 10 variables with nonzero coefficients were retained, while the coefficients of the remaining 30 variables were shrunk to zero and thus excluded from subsequent models. The selected variables included maternal age, pre-pregnancy BMI, EPDS score, place of residence, personality, per capita monthly income, number of pregnancies, mode of conception, intended pregnancy, and presence of underlying diseases. The detailed coefficients for these variables are presented in Supplementary Table S3.

### 3.3 Comparison of PBs in pregnant women with different depressive symptom status

Table 1 presents the distribution of maternal PBs exposure levels during pregnancy. The median serum concentrations of PBs ranged from 0.29 ng/mL for EtP to 4.50 ng/mL for HeP. Importantly, among mothers classified into the depressive symptoms group and the non-significant depressive symptoms group, only the EtP exposure level showed a significant difference.

**Table 1:** Concentrations (ng/mL) of five PBs in maternal serum.

PBs	% > LOD	Overall (*n* = 934)			Depressive symptoms (*n* = 418)			Non-significant depressive symptoms (*n* = 516)			*p*
		25th	50th	75th	25th	50th	75th	25th	50th	75th	
MeP	99.89	2.76	4.33	8.06	2.79	4.55	8.08	2.74	4.25	7.93	0.568
EtP	63.92	0.05	0.29	0.87	0.05	0.37	0.98	0.05	0.22	0.78	**0.015**
PrP	100	1.11	2.39	5.34	1.17	2.33	5.78	1.08	2.41	5.09	0.395
BuP	99.57	1.32	1.96	2.97	1.34	2.08	3.15	1.27	1.89	2.89	0.059
HeP	99.89	2.65	4.50	8.83	2.72	4.54	8.88	2.60	4.49	8.74	0.501

*p*-value represents the difference of maternal PBs levels between the depressive symptoms group (EPDS ≥ 9) and the non-significant depressive symptoms group (EPDS < 9). Wilcoxon rank-sum test was used to compare the difference in maternal PBs levels between the depressive symptoms group and the non-significant depressive symptoms group. Boldface indicates statistical significance (*p* < 0.05).

### 3.4 Association between prenatal PBs exposure and adverse pregnancy outcomes, and the modifying effect of depressive symptoms

**Figure 3:**
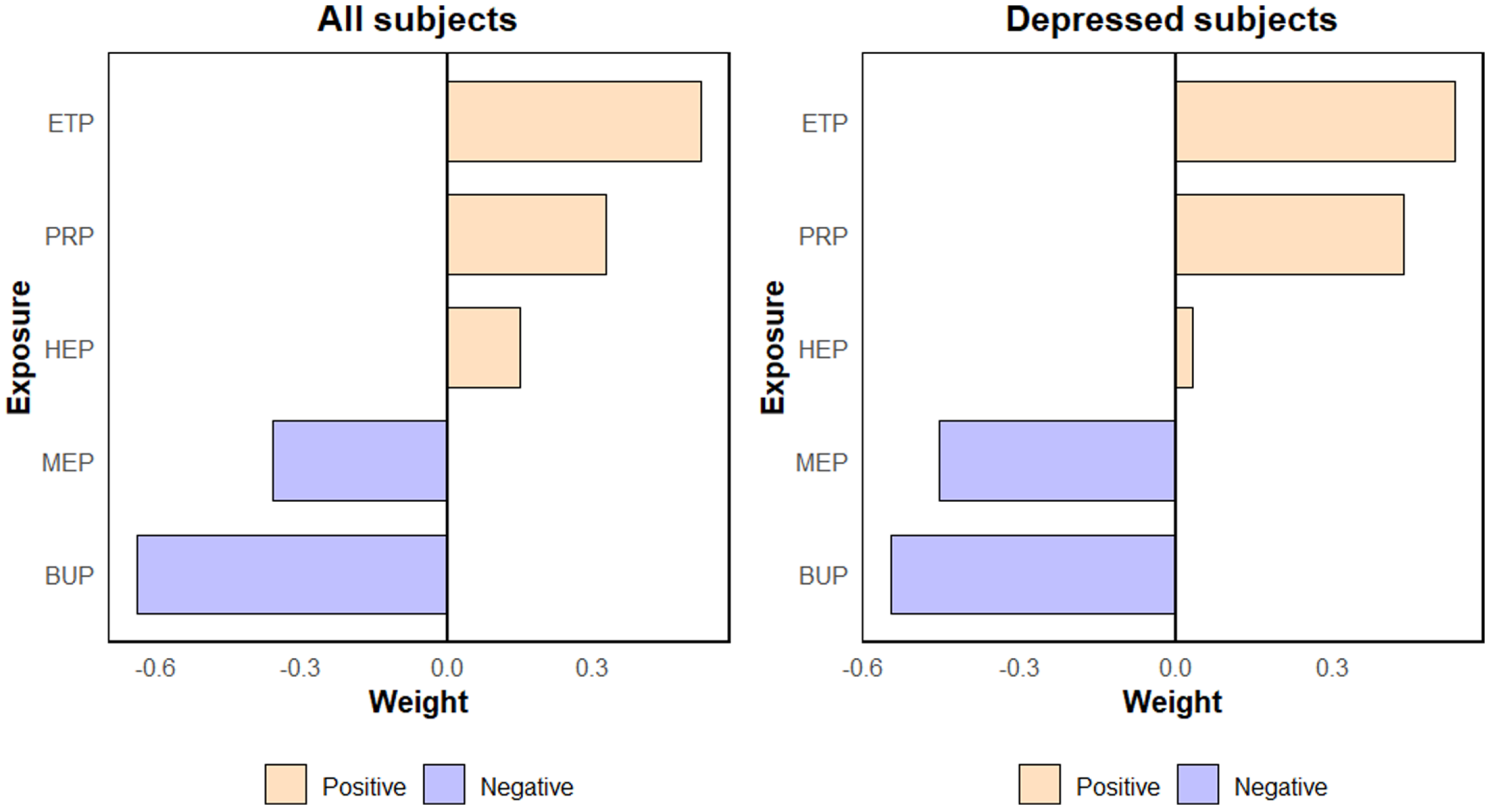
Weights of PBs for macrosomia derived from quantile g-computation in all subjects and in positive subjects.

As shown in Table 2, the associations between single and mixed PBs exposure and adverse pregnancy outcomes. Maternal serum level of PrP (OR = 1.25, 95% CI: 1.05–1.49) was positively correlated with the occurrence of fetal distress. Similarly, mixed PBs exposure was positively correlated with macrosomia. After false discovery rate (FDR) correction, none of these associations remained statistically significant at the FDR < 0.05 level, but the effect estimates and direction were largely unchanged. As shown in Figure 3, the weights of PBs compounds associated with macrosomia, derived from quantile g-computation among all subjects. The individual PBs compounds contributed differently to the combined effect, with EtP (weight = 0.53), PrP (weight = 0.33), and HeP (weight = 0.14) being the primary positive contributors. As shown in Supplementary Figure S2, the weights of PBs compounds associated with APOs, derived from quantile g-computation among all subjects, are presented. Different individual PBs compounds contributed differently to this combined effect, with EtP (weight = 0.55), MeP (weight = 0.32), PrP (weight = 0.09), and BuP (weight = 0.02) being the primary positive contributors. As shown in Supplementary Figure S3, the weights of PBs compounds associated with SVN, derived from quantile g-computation among all subjects, are presented. Different individual PBs compounds contributed variably to this combined effect, with MeP (weight = 0.76) and EtP (weight = 0.24) being the main positive contributors. As shown in Supplementary Figure S4, the weights of PBs compounds associated with fetal distress, derived from quantile g-computation among all subjects, are presented. Different individual PBs compounds contributed differently to the combined effect, with PrP (weight = 0.53), HeP (weight = 0.24), BuP (weight = 0.15), and MeP (weight = 0.08) being the primary positive contributors. As shown in Supplementary Figure S5, the weights of PBs compounds associated with asphyxia, derived from quantile g-computation among all subjects, are presented. Among the individual PBs compounds, BuP (weight = 1) stands out as the main positive contributor to this combined effect.

**Table 2:** Association between prenatal PBs exposure and adverse pregnancy outcomes.

PBs	APOs	*p*	FDR
Mixture	1.10 (0.89, 1.36)	0.367	0.551
MeP	1.10 (0.96, 1.26)	0.125	0.262
EtP	1.07 (0.98, 1.16)	0.131	0.262
PrP	1.09 (0.98, 1.20)	0.120	0.262
BuP	1.02 (0.87, 1.20)	0.837	0.930
HeP	1.01 (0.86, 1.19)	0.930	0.930

PBs	Macrosomia	*p*	FDR
Mixture	**1.62 (1.06,2.48)**	**0.027**	0.162
MeP	1.16 (0.90, 1.49)	0.246	0.369
EtP	1.15 (0.96, 1.34)	0.098	0.196
PrP	1.20 (0.97, 1.48)	0.093	0.196
BuP	1.06 (0.77, 1.46)	0.704	0.704
HeP	1.17 (0.85, 1.61)	0.340	0.408

PBs	Asphyxia	*p*	FDR
Mixture	0.78 (0.57, 1.08)	0.136	0.272
MeP	0.83 (0.66, 1.04)	0.114	0.272
EtP	0.95 (0.83, 1.08)	0.400	0.499
PrP	0.94 (0.81, 1.09)	0.416	0.499
BuP	0.83 (0.64, 1.06)	0.133	0.272
HeP	0.98 (0.77, 1.25)	0.892	0.892

PBs	SVN	*p*	FDR
Mixture	1.04 (0.80, 1.35)	0.779	0.926
MeP	1.14 (0.97, 1.35)	0.091	0.546
EtP	1.07 (0.97, 1.19)	0.190	0.570
PrP	0.99 (0.88, 1.13)	0.926	0.926
BuP	1.07 (0.88, 1.29)	0.520	0.849
HeP	0.94 (0.77, 1.16)	0.566	0.849

PBs	Fetal distress	*p*	FDR
Mixture	1.34 (0.93, 1.93)	0.118	0.354
MeP	1.14 (0.92, 1.41)	0.181	0.362
EtP	1.05 (0.91, 1.21)	0.522	0.522
PrP	**1.25 (1.05, 1.49)**	**0.012**	0.072
BuP	1.16 (0.88, 1.53)	0.284	0.426
HeP	1.10 (0.83, 1.46)	0.499	0.522

Binary logistic regression models were adjusted age, pre-pregnancy BMI, EPDS score, place of residence, personality, per capita monthly income, number of pregnancies, mode of conception, intended pregnancy, and presence of underlying diseases. Quantile g-computation was used to estimate the mixture effects of PBs. Models adjusted for age, pre-pregnancy BMI, EPDS score, place of residence, personality, per capita monthly income, number of pregnancies, mode of conception, intended pregnancy, and presence of underlying diseases. Boldface indicates statistical significance (*p* < 0.05).

As shown in Table 3, significant interaction effects were observed between maternal depressive symptoms and EtP, as well as between maternal depressive symptoms and PrP. In the depressive symptoms group, PrP had a significant positive correlation with adverse pregnancy outcomes. Specifically, for each 1-unit increase in the natural log-transformed exposure level of PrP, the risk of adverse pregnancy outcomes increased by 21% (95% CI: 1.03–1.41). For each 1-unit increase in the natural log-transformed exposure level of EtP, the risk of small and fragile newborns increased by 20% (95% CI: 1.04–1.38). In contrast, no significant correlations were observed between these compounds and adverse pregnancy outcomes or small and fragile newborns in the non-significant depressive symptoms group. Notably, the direction of EtP associations differed by depressive symptom status: EtP was positively associated with SVN in women with depressive symptoms, whereas it showed a positive association with macrosomia in women without significant depressive symptoms.

**Table 3:** Stratified analysis of associations between prenatal paraben exposure and adverse pregnancy outcomes by maternal depressive status.

PBs	APOs	*P*-int	SVN	*P*-int	Macrosomia	*P*-int	Fetal distress	*P*-int	Asphyxia	*P*-int
Depressive symptoms										
Mixture	1.15 (0.83, 1.58)		1.41 (0.96, 2.05)		1.20 (0.66, 2.19)		1.23 (0.68, 2.21)		0.81 (0.54, 1.21)	
MeP	1.09 (0.88, 1.35)	0.877	1.22 (0.97, 1.55)	0.440	1.00 (0.65, 1.52)	0.459	1.14 (0.83, 1.57)	0.972	0.83 (0.61, 1.12)	0.975
EtP	1.07 (0.95, 1.21)	0.845	**1.20 (1.04, 1.38)**	**0.024**	1.01 (0.80, 1.28)	0.169	0.96 (0.77, 1.19)	0.264	0.92 (0.79, 1.09)	0.959
PrP	**1.21 (1.03, 1.41)**	**0.049**	1.09 (0.92, 1.30)	0.114	1.15 (0.86, 1.53)	0.750	**1.33 (1.04, 1.71)**	0.547	0.99 (0.82, 1.20)	0.216
BuP	1.07 (0.83, 1.37)	0.771	1.15 (0.86, 1.52)	0.491	1.00 (0.63, 1.58)	0.816	1.25 (0.85, 1.85)	0.585	0.82 (0.59, 1.15)	0.726
HeP	1.08 (0.84, 1.37)	0.643	1.19 (0.90, 1.57)	**0.033**	1.04 (0.65, 1.66)	0.583	0.82 (0.52, 1.30)	**0.061**	1.05 (0.77, 1.42)	0.785
Non-significant depressive symptoms										
Mixture	1.10 (0.82, 1.49)		0.71 (0.48, 1.05)		**2.30 (1.21, 4.36)**		1.63 (0.97, 2.72)		0.80 (0.44, 1.45)	
MeP	1.13 (0.94, 1.36)		1.07 (0.86, 1.34)		1.28 (0.91, 1.81)		1.12 (0.83, 1.52)		0.87 (0.59, 1.29)	
EtP	1.06 (0.94, 1.19)		0.94 (0.81, 1.10)		**1.28 (1.02, 1.61)**		1.12 (0.93, 1.36)		0.96 (0.77, 1.20)	
PrP	0.98 (0.85, 1.14)		0.91 (0.76, 1.09)		1.24 (0.91, 1.67)		1.17 (0.92, 1.50)		0.83 (0.63, 1.08)	
BuP	1.03 (0.83, 1.27)		1.01 (0.77, 1.31)		1.13 (0.72, 1.77)		1.08 (0.75, 1.57)		0.90 (0.59, 1.36)	
HeP	1.00 (0.80, 1.25)		**0.73 (0.54, 0.99)**		1.36 (0.87, 2.12)		1.42 (0.99, 2.04)		0.96(0.63,1.46)	

*P*-int: *p* value for interaction. Binary logistic regression models or quantile g-computation models were adjusted for age, pre-pregnancy BMI, EPDS score, place of residence, personality, per capita monthly income, number of pregnancies, mode of conception, intended pregnancy, and presence of underlying diseases. Boldface indicates statistically significant associations (95% CI not crossing 1) or suggestive interactions (*P*-int < 0.1).

### 3.5 Sensitivity analysis

To evaluate the robustness of the main findings of this study, we conducted a sensitivity analysis by excluding pregnant women with a history of more than one spontaneous abortion and repeated the primary statistical analysis procedures.

As shown in Supplementary Table S3, the results of both binary logistic regression and quantile g-computation (qgcomp) models were largely consistent with those of the primary analysis. Although the overall mixture of PBs showed no significant association with APOs (OR = 1.11, 95% CI: 0.88–1.40), a significant positive association was observed with macrosomia (OR = 1.91, 95% CI: 1.21–3.04). As shown in Supplementary Figure S6–S10, the mixture weights of PBs derived from the qgcomp model—reflecting the direction and relative contribution of each pollutant to the joint effect—were consistent with those of the primary analysis across all outcomes in the sensitivity analysis.

As shown in Supplementary Table S4, the stratified analysis based on maternal depressive symptoms further revealed certain effect modification patterns. The association between PrP and APOs was significantly modified by maternal depressive symptoms (*P*-int = 0.045), with a similarly strong positive association observed in women with depressive symptoms (OR = 1.23, 95% CI: 1.04–1.46). Additionally, the PBs mixture was significantly associated with SVN in women with depressive symptoms (OR = 1.51, 95% CI: 1.00–2.26).

In conclusion, this sensitivity analysis confirms that the main findings of this study regarding the associations between prenatal paraben exposure and specific adverse pregnancy outcomes, as well as the effect modification by maternal depressive status, are robust and not significantly influenced by a history of multiple spontaneous abortions.

## 4 Discussion

In this study, we investigated the individual and joint associations of prenatal PBs exposure with adverse pregnancy outcomes, with a particular focus on whether maternal depressive symptoms modify these relationships. Our results indicated that specific PBs congeners, particularly PrP and EtP, were associated with an increased risk of certain adverse pregnancy outcomes, and these associations were significantly modulated by maternal psychological status.

Consistent with previous studies, we observed a positive correlation between PrP and fetal distress, while mixed preservative exposure was associated with an increased risk of macrosomia. For example, a prospective cohort study involving 529 mother-infant pairs found that exposure to MeP in the urine of pregnant women during mid-pregnancy was negatively associated with a reduction in birth weight in female offspring, but the association was not statistically significant; at the same time, exposure to butylparaben was also related to a smaller birth weight in female offspring (23). In another large-scale prospective study covering 3,619 mother-infant pairs, exposure to benzophenone-3 and methylparaben during pregnancy was associated with lower birth weight, lower birth weight after gestational age adjustment, and an increased risk of small-for-gestational-age infants (8). In addition, a study of 483 mother–infant pairs reported that prenatal exposure to a mixture of parabens and other chemicals was significantly associated with reduced birth weight, birth length, head circumference, and chest circumference in newborns (24). Mechanistic evidence also supports these findings: a study of pregnant women in Puerto Rico found that prenatal exposure to parabens (such as methylparaben) and other chemicals (such as bisphenols and triclosan) was significantly associated with changes in inflammatory markers in pregnant women (such as CRP, matrix metalloproteinases, and cell adhesion molecules), and these changes in inflammatory markers may increase the risk of adverse pregnancy outcomes, such as preterm birth and preeclampsia. Moreover, the impact of these chemicals on inflammatory markers may vary depending on fetal sex and timing of exposure, and may have potential long-term health effects on offspring, suggesting that prenatal exposure to parabens not only affects inflammatory responses during pregnancy but may also have adverse effects on the long-term health of offspring (25). Complementing these findings, our quantile g-computation results further identified EtP, PrP, and HeP as the main contributors to the mixture effect on macrosomia, underscoring the importance of considering co-exposure effects when assessing the health impacts of environmental chemicals.

More importantly, we identified a significant interaction between maternal depressive symptoms and PBs exposure. In the depressive symptoms group, PrP exposure was associated with an increased risk of overall APOs, while EtP was associated with a higher risk of SVN; by contrast, these significant associations were not observed in the non-significant depressive symptoms group. To verify the robustness of these findings, we conducted a sensitivity analysis excluding women with a history of multiple spontaneous abortions; the results were essentially consistent with the main analysis, and the quantile g-computation weights remained stable across subsamples, confirming the reliability of the observed mixture effects. The interaction between depressive symptoms and PB exposure may be explained by shared biological pathways, particularly inflammatory responses and hypothalamic–pituitary–adrenal (HPA) axis dysregulation. For example, exposure to parabens during pregnancy can significantly reduce cortisol levels, with this hormonal imbalance being more pronounced in female fetuses (26), and is associated with an increased risk of pregnancy-induced hypertension (27). Concurrently, maternal depressive symptoms, as an independent risk factor, may interact with environmental exposures (such as air pollutants) to predict adverse outcomes through inflammation-mediated mechanisms (28). Existing evidence supports that inflammatory pathways (such as NF-κB-mediated pro-inflammatory gene expression) and HPA axis hormonal dysregulation are core biological mechanisms. These two factors may interact synergistically to exacerbate the maternal stress response and increase inflammation levels, thereby jointly promoting adverse pregnancy outcomes (28–30).

The finding that depressive symptoms increase susceptibility to environmental exposures is consistent with multiple previous studies. For example, studies have shown that prenatal depressive symptoms can amplify the impact of air pollutants (such as particulate matter, nitrogen dioxide, and ozone) on the risk of pregnancy-induced hypertension, an effect not observed in women without significant depressive symptoms (31). Furthermore, research indicates that prenatal environmental exposures and maternal mental health interact to influence the long-term health of offspring. For instance, studies have found that under early nitrogen oxide exposure trajectories, maternal depressive symptoms exacerbates offspring’s emotional disorders, psychiatric symptoms, and accelerates brain aging (32). Prenatal exposure to environmental chemicals such as phthalates and bisphenols is not only significantly associated with an increased risk of postpartum depression and anxiety symptoms (33, 34), but when these chemicals are mixed with psychosocial stress, they also have a direct negative impact on pregnancy outcomes, such as reduced birth weight (35). Together, these findings indicate that psychological stress may act as a key modifier of environmental health risks across multiple chemicals and health outcomes, supporting the interaction observed between maternal depressive symptoms and PBs exposure.

In addition, an unexpected but noteworthy finding is the divergent association of EtP with different APOs depending on depressive symptom status. Among women with depressive symptoms, EtP was associated with an increased risk of SVN, whereas in women without significant depressive symptoms, EtP showed a positive association with macrosomia. Although seemingly contradictory, this pattern is biologically plausible and underscores the critical role of maternal psychological state in modifying the effects of environmental chemical exposures.

First, parabens have been reported to exhibit obesogenic potential. Experimental studies suggest that several parabens can promote adipocyte differentiation and may activate nuclear receptors such as peroxisome proliferator-activated receptor gamma (PPARγ), thereby influencing lipid metabolism. These effects may contribute to altered fetal growth patterns, particularly in metabolically favorable conditions (36, 37). This aligns with the positive association between EtP and macrosomia observed in women without significant depressive symptoms. Second, parabens including EtP can cross the placental barrier, with studies showing measurable fetal-to-maternal concentration ratios in paired maternal and cord serum samples, and ex-vivo human placental perfusion models indicating near-equivalent transfer for EtP and other parabens (10, 38). In the context of maternal depressive symptoms—characterized by HPA axis dysregulation, elevated glucocorticoid levels, and heightened inflammatory status—placental handling of xenobiotics may be substantially altered, potentially shifting the net effect toward restricted fetal growth. Third, emerging evidence suggests that the metabolic effects of environmental chemicals may vary depending on underlying physiological and psychosocial conditions. Several studies highlight that environmental chemical exposures and maternal stressors may interact to influence perinatal outcomes, potentially altering biological pathways such as HPA-axis regulation, inflammation, and nutrient transport. For example, while certain EDCs may promote adipogenesis under baseline conditions, their effects in the presence of heightened maternal stress and altered hormonal responses could shift fetal development trajectories, including impaired nutrient transfer and growth patterns (39). Collectively, these mechanisms support the observation that the same chemical exposure may yield divergent fetal outcomes depending on maternal psychological status, highlighting the importance of considering both environmental and psychosocial factors in prenatal risk assessment.

In China, parabens are primarily used in personal care products, cosmetics, and packaged foods, with MeP and PrP being the most frequently detected congeners in the general population. Our findings suggest that reducing unnecessary exposure to paraben-containing products during pregnancy, particularly among women with depressive symptoms, may represent a feasible public health strategy. Routine antenatal care could incorporate depressive symptom screening to identify high-risk subgroups, allowing for targeted guidance on environmental exposure reduction.

It is noteworthy that the proportion of women with depressive symptoms (EPDS ≥ 9) in this study was 44.8%, which is higher than the 25.8% reported in a recent large-scale national survey of 100,020 pregnant women across 11 provinces in China (40). Several factors may explain this relatively higher screening rate. First, participants were recruited from a single tertiary hospital, where pregnant women are more likely to experience high-risk pregnancies, pregnancy complications, or increased health-related concerns, all of which may contribute to elevated psychological distress. By contrast, national surveys often include participants from both community and hospital settings, resulting in lower overall prevalence estimates. Second, it is important to emphasize that the EPDS is a screening tool rather than a diagnostic instrument. It is designed to maximize sensitivity and identify individuals at risk of depression. Previous validation studies in Chinese populations have shown that although a substantial proportion of pregnant women may screen positive (EPDS ≥ 9), the prevalence of clinically diagnosed depression is considerably lower. For instance, evidence from a large individual participant data meta-analysis indicates that estimates of depression prevalence based on the EPDS (e.g., EPDS ≥ 9) tend to be substantially higher than diagnoses confirmed by structured clinical interview; pooled EPDS ≥ 9 prevalence was around 27.8%, while SCID-based major depression prevalence was approximately 9.0%. This highlights that the EPDS primarily identifies depressive symptoms rather than clinical diagnoses (41). Therefore, the prevalence reported in this study reflects depressive symptoms rather than clinically diagnosed depression, which may partly explain the higher observed rate. Third, differences in study setting, socioeconomic factors, and the timing of assessment may also contribute to variability in prevalence estimates across studies. Importantly, the primary objective of this study was not to estimate the population prevalence of depressive symptoms, but to evaluate their effect-modifying role in the association between prenatal PBs exposure and APOs. The internal comparison between women with and without depressive symptoms within the same cohort remains valid regardless of the absolute prevalence. Therefore, although caution is warranted when generalizing the absolute prevalence estimate to the broader population, the internal validity of the observed interaction effects is unlikely to be compromised, and these findings provide meaningful insights into the joint effects of environmental and psychosocial exposures during pregnancy.

The strengths of this study lie in its comprehensive approach to examining the interaction between prenatal paraben exposure and maternal depressive symptoms on adverse pregnancy outcomes. As a prospective cohort study, we were able to establish a temporal relationship between exposure and outcomes, which strengthens the causal inference. By utilizing a large sample size and rigorous statistical methods, including LASSO regression and quantile g-computation, we were able to control for multiple covariates and assess the combined effects of paraben mixtures. Additionally, the use of the EPDS provided a validated tool for assessing maternal psychological distress. However, several limitations should be acknowledged. Although we controlled for several covariates, there may be other unmeasured confounders that could influence the results. For example, we did not assess the specific sources of paraben exposure, such as diet, personal care products, or environmental factors, which may lead to exposure misclassification and limit our ability to identify key exposure pathways. Additionally, the EPDS is a self-report questionnaire, and while it is widely used, it may not capture all aspects of psychological distress during pregnancy. Future research should focus on longitudinal studies with detailed exposure assessments and multiple measurement time points to better understand the dynamics of exposure and outcomes. Moreover, experimental designs or interventions could help elucidate the underlying biological mechanisms of the observed interactions.

## 5 Conclusion

In this prospective cohort study, prenatal PBs exposure was associated with an increased risk of APOs, with PrP significantly linked to fetal distress and mixed exposure associated with elevated risk of macrosomia. More importantly, maternal depressive symptoms exhibited a significant effect modification on these associations, where the impacts of PrP and EtP on APOs were more pronounced among women with depressive symptoms. These findings highlight the necessity of integrating both environmental chemical exposure and mental health status into prenatal care to better identify high-risk populations and develop comprehensive intervention strategies.

## Data Availability

The original contributions presented in the study are included in the article/supplementary material, further inquiries can be directed to the corresponding author/s.
