# Supplementary material for: Association of prenatal parabens exposure with adverse pregnancy outcomes: the role of prenatal depressive symptoms
Source: Front Public Health. 2026 Apr 23;14:1802833. doi: 10.3389/fpubh.2026.1802833 (PMC13149364; doi:10.3389/fpubh.2026.1802833)
Supplement: Supplementary file 1 [file Supplementary_file_1.DOCX]

Supplementary Material

# Supplementary Figures and Tables

## Supplementary Figures


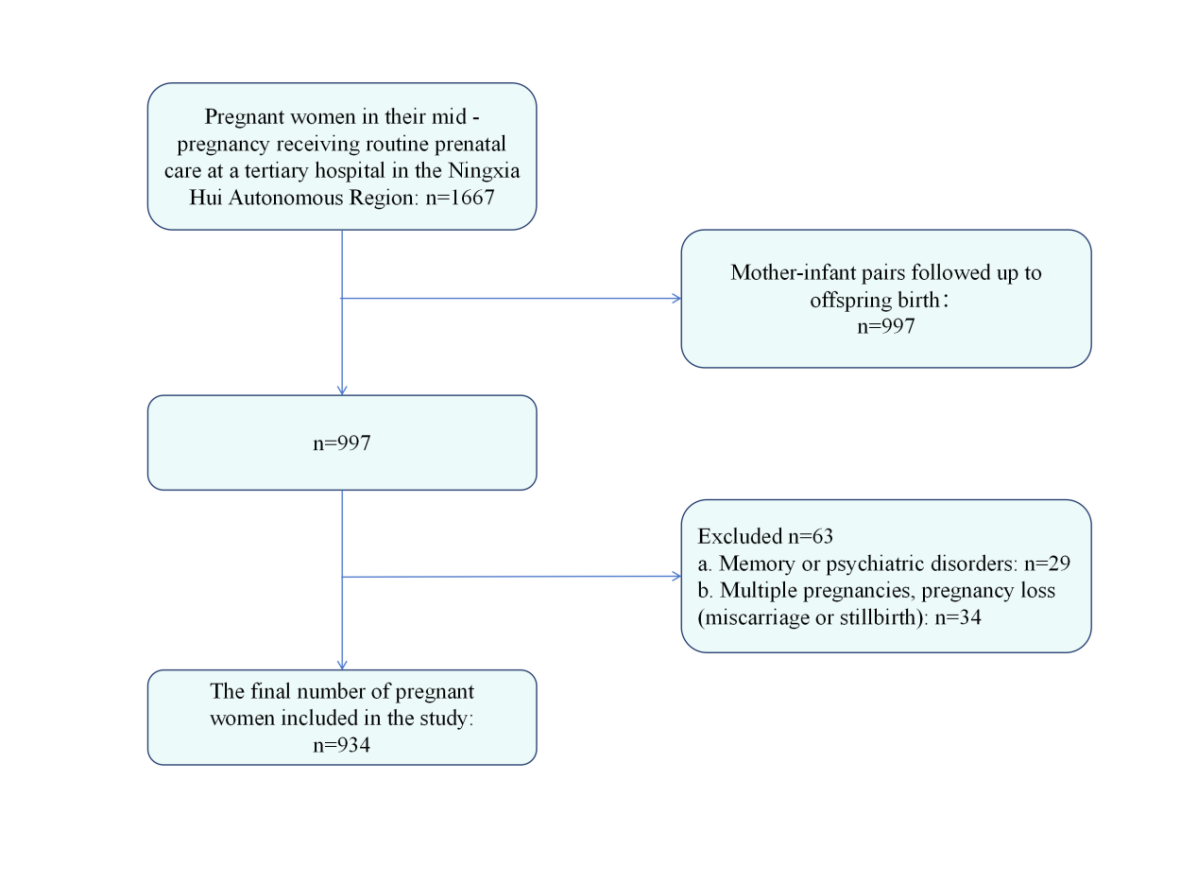


**Supplementary Figure 1.** Flow chart of participant selection in the study


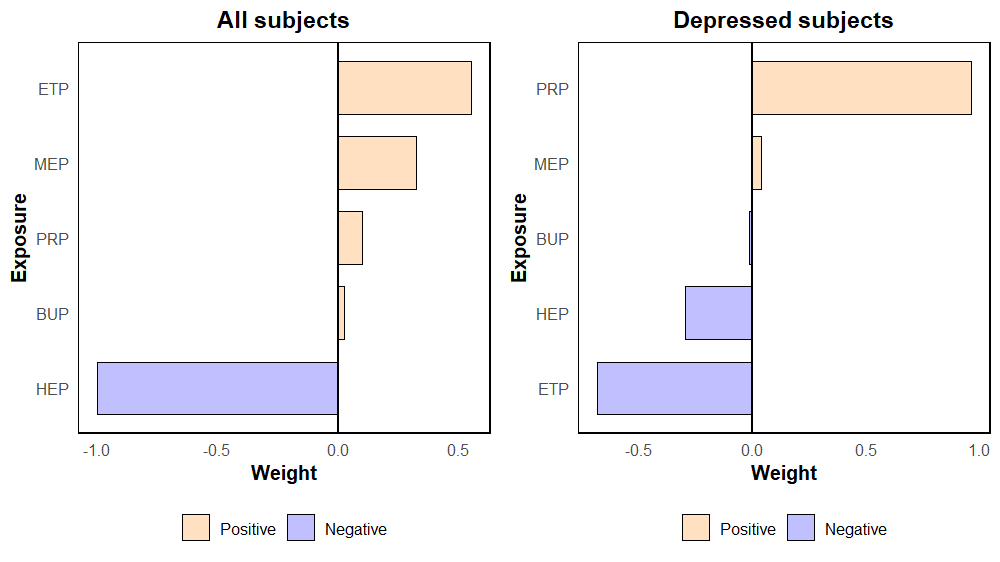


**Supplementary Figure 2.** Weights of PBs for APOs derived from quantile g-computation in all subjects and in women with depressive symptoms.


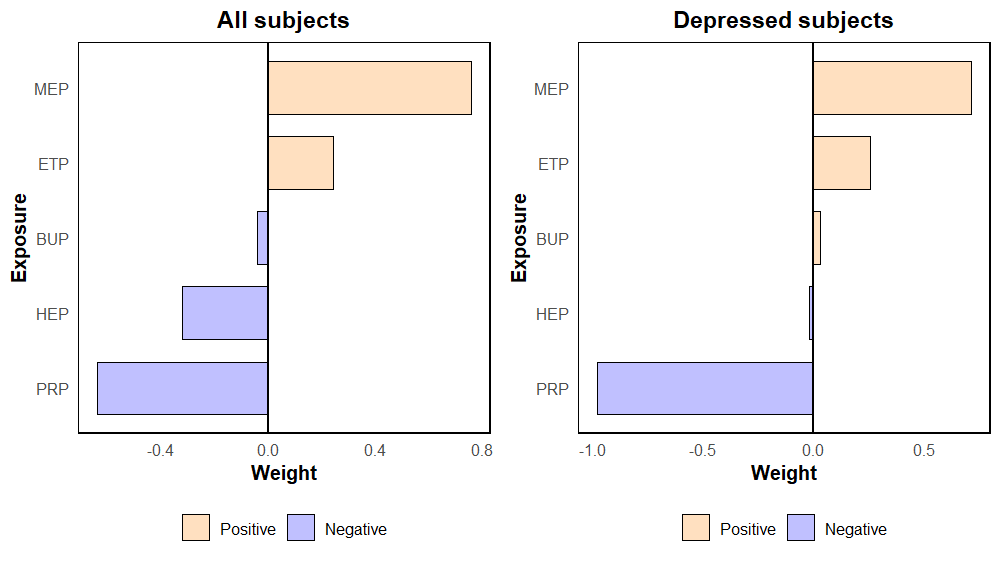
**Supplementary Figure 3.** Weights of PBs for SVN derived from quantile g-computation in all subjects and in women with depressive symptoms.


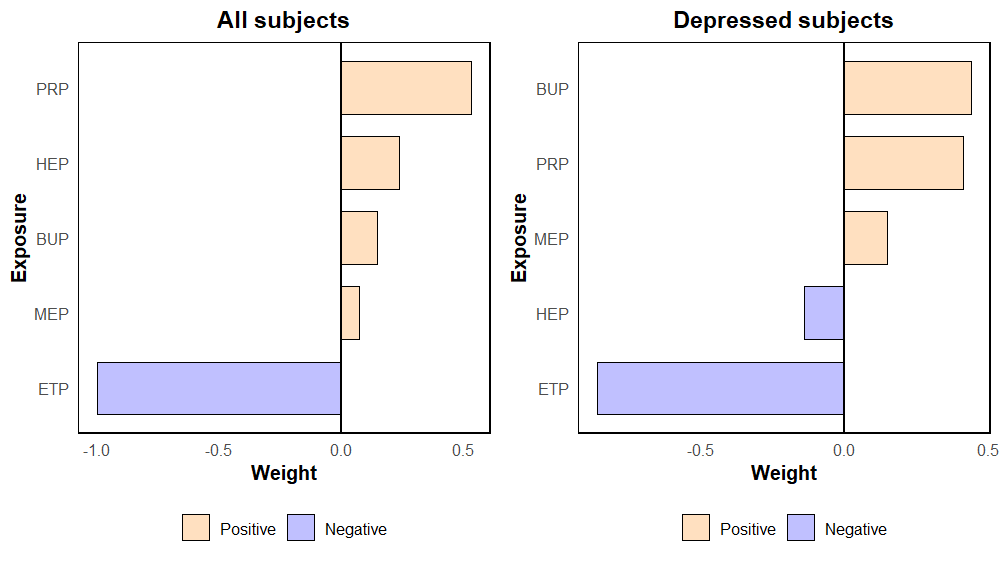


**Supplementary Figure 4.** Weights of PBs for Fetal distress derived from quantile g-computation in all subjects and in women with depressive symptoms.


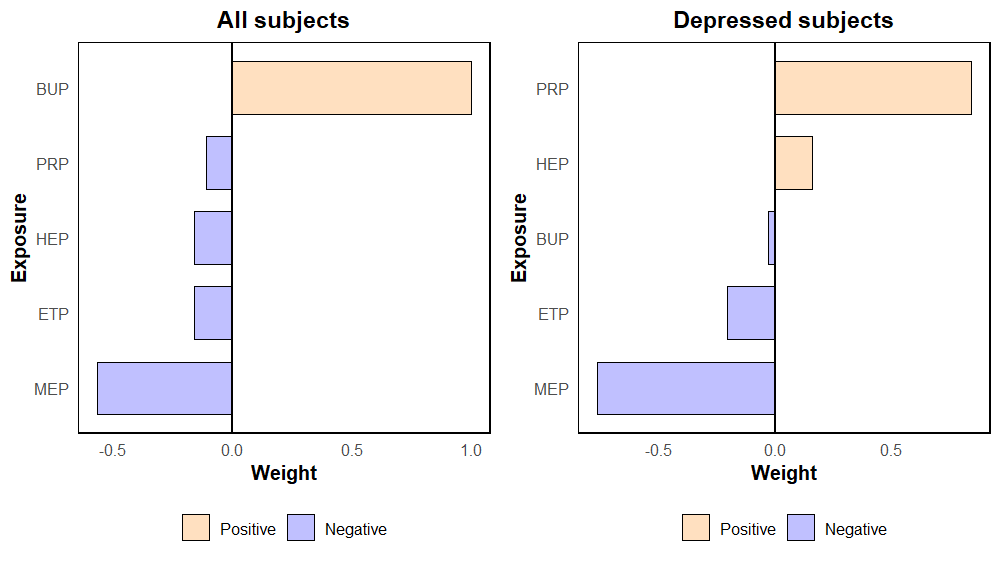


**Supplementary Figure 5.** Weights of PBs for Asphyxia derived from quantile g-computation in all subjects and in women with depressive symptoms.


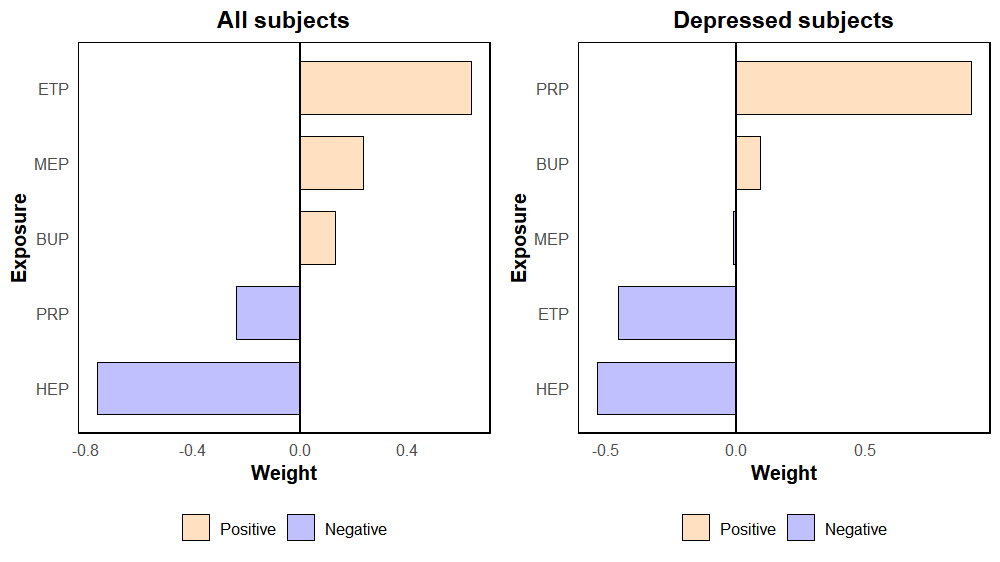


**Supplementary Figure 6.** Weights of PBs for APOs from quantile g-computation in all subjects and in women with depressive symptoms.


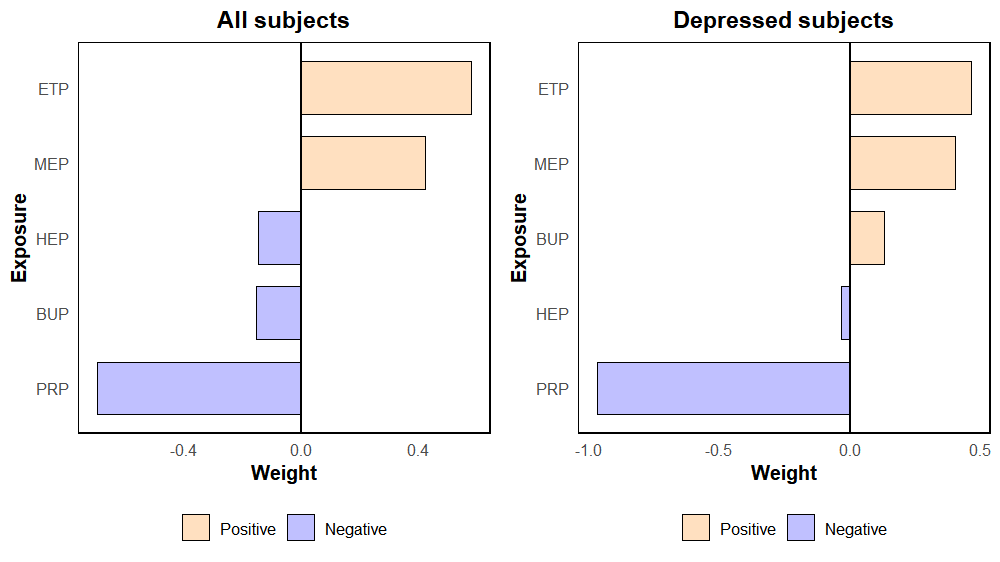


**Supplementary Figure 7.** Weights of PBs for SVN from quantile g-computation in all subjects and in women with depressive symptoms.


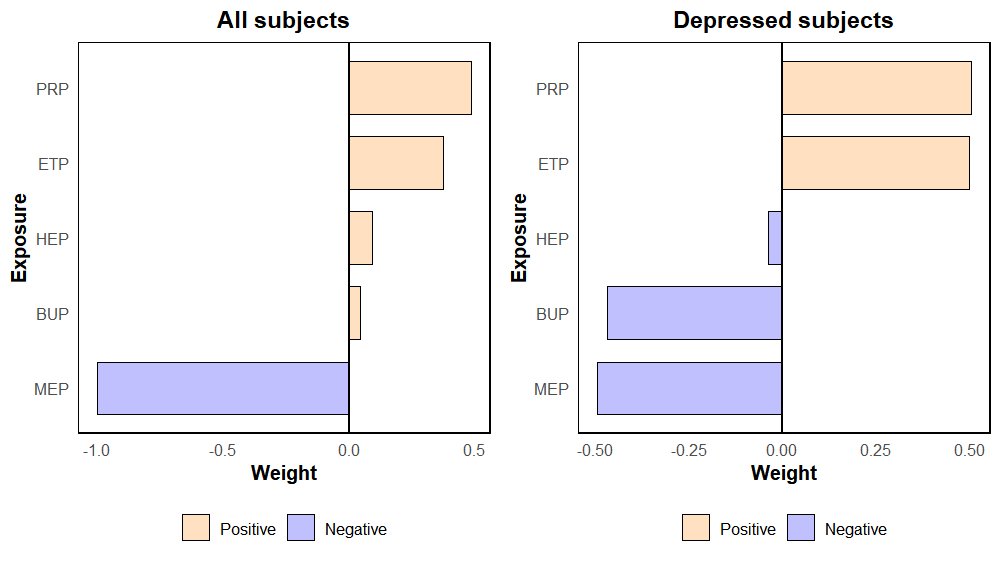


**Supplementary Figure 8.** Weights of PBs for Macrosomia from quantile g-computation in all subjects and in women with depressive symptoms.


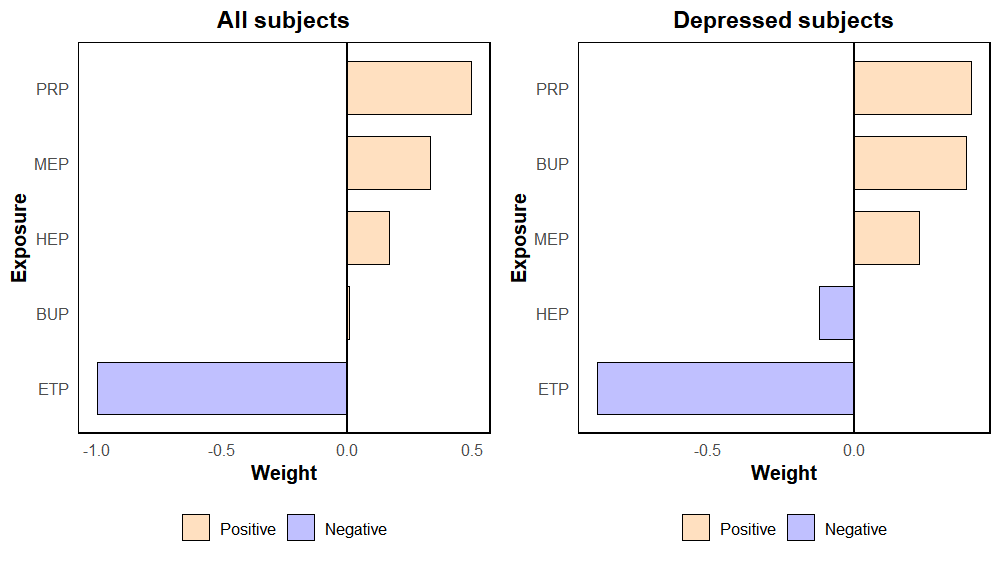


**Supplementary Figure 9.** Weights of PBs for Fetal distress from quantile g-computation in all subjects and in women with depressive symptoms.


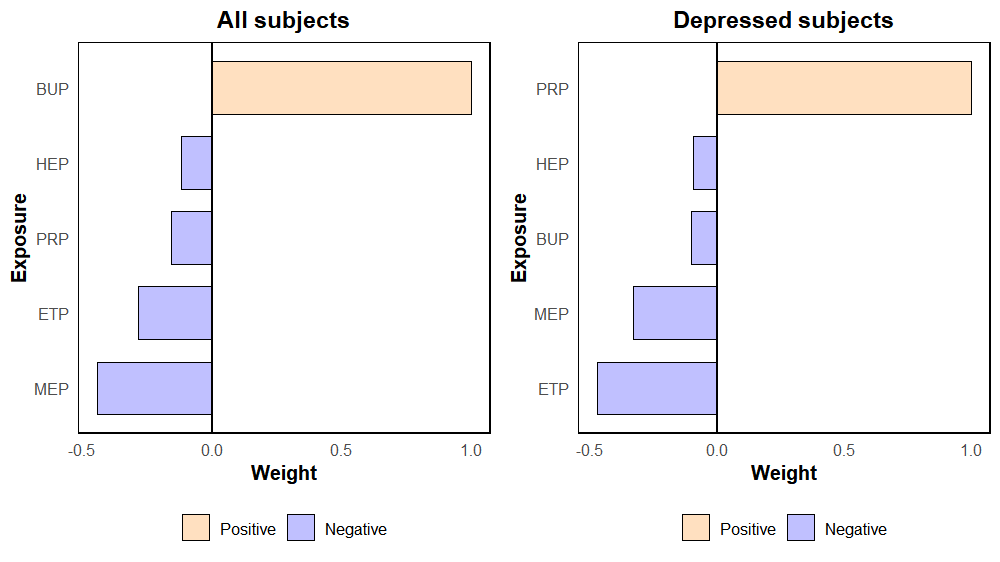


**Supplementary Figure 10.** Weights of PBs for Asphyxia from quantile g-computation in all subjects and in women with depressive symptoms.

## Supplementary Tables

**Supplementary Table S1.** Demographic Characteristics of the Study Participants (Mothers)

| Characteristics | Participants, Frequency (N) (proportion, %) | | | Statistic | *P* |
| --- | --- | --- | --- | --- | --- |
|  | Overall (n = 934) | Non‑significant depressive symptoms (n = 516) | Depressive symptoms  (n = 418) |  |  |
| Number of pregnancies, M (Q₁, Q₃) | 2.00 (1.00, 3.00) | 2.00 (1.00, 3.00) | 2.00 (1.00, 3.00) | Z=-0.80 | 0.423 |
| Maternal age (years) |  |  |  | *χ²*=1.12 | 0.289 |
| <35 | 766 (82.01) | 417 (80.81) | 349 (83.49) |  |  |
| ≥35 | 168 (17.99) | 99 (19.19) | 69 (16.51) |  |  |
| Pre-pregnancy BMI (kg/m^2^) |  |  |  | *χ²*=0.99 | 0.608 |
| <25 | 752 (80.51) | 410 (79.46) | 342 (81.82) |  |  |
| 25-30 | 137 (14.67) | 81 (15.70) | 56 (13.40) |  |  |
| >30 | 45 (4.82) | 25 (4.84) | 20 (4.78) |  |  |
| Place of residence |  |  |  | *χ²*=4.96 | **0.026** |
| Urban area | 812 (86.94) | 460 (89.15) | 352 (84.21) |  |  |
| Rural area | 122 (13.06) | 56 (10.85) | 66 (15.79) |  |  |
| Maternal education |  |  |  | *χ²*=3.71 | 0.294 |
| Junior high school or below | 109 (11.67) | 68 (13.18) | 41 (9.81) |  |  |
| High school / Vocational high school | 112 (11.99) | 66 (12.79) | 46 (11.00) |  |  |
| Associate degree / Bachelor's degree | 664 (71.09) | 355 (68.80) | 309 (73.92) |  |  |
| Graduate degree or above | 49 (5.25) | 27 (5.23) | 22 (5.26) |  |  |
| Occupation |  |  |  | *χ²*=4.87 | 0.181 |
| Unemployed | 209 (22.38) | 121 (23.45) | 88 (21.05) |  |  |
| Farmer / Worker | 26 (2.78) | 17 (3.29) | 9 (2.15) |  |  |
| Administrative Unit | 344 (36.83) | 175 (33.91) | 169 (40.43) |  |  |
| Other | 355 (38.01) | 203 (39.34) | 152 (36.36) |  |  |
| Personality |  |  |  | *χ²*=12.53 | **0.002** |
| Introverted | 134 (14.35) | 61 (11.82) | 73 (17.46) |  |  |
| Ambiverted | 619 (66.27) | 337 (65.31) | 282 (67.46) |  |  |
| Extroverted | 181 (19.38) | 118 (22.87) | 63 (15.07) |  |  |
| Whether the mother was an only child |  |  |  | *χ²*=2.01 | 0.156 |
| Yes | 135 (14.45) | 67 (12.98) | 68 (16.27) |  |  |
| No | 799 (85.55) | 449 (87.02) | 350 (83.73) |  |  |
| Ethnicity |  |  |  | χ²=1.79 | 0.181 |
| Han Chinese | 738 (79.01) | 416 (80.62) | 322 (77.03) |  |  |
| Other | 196 (20.99) | 100 (19.38) | 96 (22.97) |  |  |
| Per capita monthly income |  |  |  | *χ²*=1.81 | 0.405 |
| <¥2000 | 44 (4.71) | 28 (5.43) | 16 (3.83) |  |  |
| ¥2000-¥5000 | 480 (51.39) | 258 (50.00) | 222 (53.11) |  |  |
| >¥5000 | 410 (43.90) | 230 (44.57) | 180 (43.06) |  |  |
| Number of spontaneous abortions |  |  |  | *χ²*=0.96 | 0.618 |
| 0 | 793 (84.90) | 443 (85.85) | 350 (83.73) |  |  |
| 1 | 110 (11.78) | 56 (10.85) | 54 (12.92) |  |  |
| ≥2 | 31 (3.32) | 17 (3.29) | 14 (3.35) |  |  |
| Number of induced abortions |  |  |  | *χ²*=1.43 | 0.490 |
| 0 | 687 (73.55) | 373 (72.29) | 314 (75.12) |  |  |
| 1 | 182 (19.49) | 103 (19.96) | 79 (18.90) |  |  |
| ≥2 | 65 (6.96) | 40 (7.75) | 25 (5.98) |  |  |
| Number of induced abortions due to fetal anomalies |  |  |  | *χ²*=0.32 | 0.853 |
| 0 | 838 (89.72) | 462 (89.53) | 376 (89.95) |  |  |
| 1 | 85 (9.10) | 47 (9.11) | 38 (9.09) |  |  |
| ≥2 | 11 (1.18) | 7 (1.36) | 4 (0.96) |  |  |
| Mode of conception |  |  |  | *χ²*=1.17 | 0.279 |
| Natural conception | 845 (90.47) | 462 (89.53) | 383 (91.63) |  |  |
| Assisted reproductive technology | 89 (9.53) | 54 (10.47) | 35 (8.37) |  |  |
| Severity of early pregnancy reactions |  |  |  | *χ²*=14.06 | **< 0.001** |
| Mild | 338 (36.19) | 204 (39.53) | 134 (32.06) |  |  |
| Moderate | 410 (43.90) | 231 (44.77) | 179 (42.82) |  |  |
| Severe | 186 (19.91) | 81 (15.70) | 105 (25.12) |  |  |
| Intended pregnancy |  |  |  | *χ²*=4.42 | 0.109 |
| Planned pregnancy | 402 (43.04) | 231 (44.77) | 171 (40.91) |  |  |
| Natural conception | 408 (43.68) | 227 (43.99) | 181 (43.30) |  |  |
| Unplanned pregnancy | 124 (13.28) | 58 (11.24) | 66 (15.79) |  |  |
| Mother's gender preference for the fetus |  |  |  | *χ²*=1.33 | 0.515 |
| Male | 56 (6.00) | 28 (5.43) | 28 (6.70) |  |  |
| Female | 106 (11.35) | 55 (10.66) | 51 (12.20) |  |  |
| Fair | 772 (82.66) | 433 (83.91) | 339 (81.10) |  |  |
| Family's gender preference for the fetus |  |  |  | *χ²*=0.31 | 0.858 |
| Male | 60 (6.42) | 36 (6.33) | 24 (6.58) |  |  |
| Female | 80 (8.57) | 51 (8.96) | 29 (7.95) |  |  |
| Fair | 794 (85.01) | 482 (84.71) | 312 (85.48) |  |  |
| Pre-pregnancy health status |  |  |  | *χ²*=32.17 | **< 0.001** |
| Good | 621 (66.49) | 381 (73.84) | 240 (57.42) |  |  |
| Moderate | 300 (32.12) | 133 (25.78) | 167 (39.95) |  |  |
| Poor | 13 (1.39) | 2 (0.39) | 11 (2.63) |  |  |
| Presence of underlying diseases |  |  |  | *χ²*=1.30 | 0.254 |
| No | 830 (88.87) | 464 (89.92) | 366 (87.56) |  |  |
| Yes | 104 (11.13) | 52 (10.08) | 52 (12.44) |  |  |
| PSQI |  |  |  | *χ²*=33.19 | **< 0.001** |
| Good | 552 (59.10) | 348 (67.44) | 204 (48.80) |  |  |
| Poor | 382 (40.90) | 168 (32.56) | 214 (51.20) |  |  |

Differences in bold are statistically significant.

**Supplementary Table S2.** Demographic Characteristics of the Study Participants (Offspring)

| Characteristics | Participants, Frequency (N) (proportion, %) | | | Statistic | *P* |
| --- | --- | --- | --- | --- | --- |
|  | Overall  (n = 934) | Non‑significant depressive symptoms  (n = 516) | Depressive symptoms  (n = 418) |  |  |
| Mode of delivery |  |  |  | *χ²*=0.61 | 0.434 |
| Vaginal delivery | 398 (42.61) | 214 (41.47) | 184 (44.02) |  |  |
| Cesarean section | 536 (57.39) | 302 (58.53) | 234 (55.98) |  |  |
| Gestational age at birth (weeks) |  |  |  | *χ²*=0.79 | 0.374 |
| ≥37 | 836 (89.51) | 466 (90.31) | 370 (88.52) |  |  |
| <37 | 98 (10.49) | 50 (9.69) | 48 (11.48) |  |  |
| Fetal Sex |  |  |  | *χ²=0.01* | 0.919 |
| Male | 500 (53.53) | 277 (53.68) | 223 (53.35) |  |  |
| Female | 434 (46.47) | 239 (46.32) | 195 (46.65) |  |  |
| Birth weight (g) |  |  |  | *χ²=4.70* | **0.030** |
| <2500 | 70 (7.49) | 30 (5.81) | 40 (9.57) |  |  |
| ≥2500 | 864 (92.51) | 486 (94.19) | 378 (90.43) |  |  |
| Birth body length (cm) |  |  |  | *χ²=2.32* | 0.313 |
| Normal | 674 (72.16) | 382 (74.03) | 292 (69.86) |  |  |
| Short | 226 (24.20) | 118 (22.87) | 108 (25.84) |  |  |
| Long | 34 (3.64) | 16 (3.10) | 18 (4.31) |  |  |
| Adverse pregnancy outcomes |  |  |  | *χ²=14.85* | **< 0.001** |
| No | 481 (51.50) | 295 (57.17) | 186 (44.50) |  |  |
| Yes | 453 (48.50) | 221 (42.83) | 232 (55.50) |  |  |

**Supplementary Table S3.** Coefficients and Lambda. 1se Values of the LASSO regression

| Variables | Coefficients | lambda.1se |
| --- | --- | --- |
| maternal age | 0.003920813 | 0.03801306 |
| pre-pregnancy BMI | 0.003707251 |  |
| EPDS score | 0.065964376 |  |
| place of residence | -0.064850871 |  |
| personality | -0.180385512 |  |
| per capita monthly income | 0.124212882 |  |
| number of pregnancies | 0.032008267 |  |
| mode of conception | 0.749750422 |  |
| intended pregnancy | 0.086291856 |  |
| presence of underlying diseases | 0.351212416 |  |

**Supplementary Table S4.** Association between prenatal PBs exposure and adverse pregnancy outcomes

| **PBs** | **APOs** | ***P*** | **FDR** |  | **SVN** | ***P*** | **FDR** |
| --- | --- | --- | --- | --- | --- | --- | --- |
| Mixture | 1.11(0.88,1.40) | 0.399 | 0.598 |  | 1.08(0.80,1.44) | 0.617 | 0.736 |
| MeP | 1.07(0.92,1.25) | 0.264 | 0.528 |  | 1.12(0.93,1.35) | 0.204 | 0.612 |
| EtP | 1.08(0.99,1.19) | 0.096 | 0.408 |  | 1.09(0.98,1.22) | 0.127 | 0.612 |
| PrP | 1.09(0.97,1.22) | 0.136 | 0.408 |  | 0.98(0.85,1.12) | 0.736 | 0.736 |
| BuP | 1.01(0.84,1.21) | 0.949 | 0.949 |  | 1.09(0.87,1.37) | 0.458 | 0.736 |
| HeP | 0.98(0.82,1.18) | 0.863 | 0.949 |  | 0.96(0.76,1.21) | 0.701 | 0.736 |
|  |  |  |  |  |  |  |  |
| **PBs** | **Macrosomia** | ***P*** | **FDR** |  | **Fetal distress** | ***P*** | **FDR** |
| Mixture | **1.91(1.21,3.04)** | **0.006** | **0.018** |  | 1.15(0.78,1.71) | 0.479 | 0.822 |
| MeP | 1.26(0.94,1.65) | 0.081 | 0.121 |  | 1.06(0.84,1.34) | 0.548 | 0.822 |
| EtP | **1.20(1.01,1.42)** | **0.044** | 0.088 |  | 0.99(0.84,1.15) | 0.854 | 0.994 |
| PrP | **1.39(1.11,1.74)** | **0.004** | **0.018** |  | 1.14(0.95,1.37) | 0.174 | 0.822 |
| BuP | 1.22(0.88,1.70) | 0.237 | 0.284 |  | 0.87(0.63,1.20) | 0.398 | 0.822 |
| HeP | 1.17(0.83,1.67) | 0.373 | 0.373 |  | 1.00(0.74,1.36) | 0.994 | 0.994 |
|  |  |  |  |  |  |  |  |
| **PBs** | **Asphyxia** | ***P*** | **FDR** |  |  |  |  |
| Mixture | 0.75(0.52,1.08) | 0.124 | 0.462 |  |  |  |  |
| MeP | 0.82(0.64,1.07) | 0.154 | 0.462 |  |  |  |  |
| EtP | 0.97(0.84,1.11) | 0.626 | 0.751 |  |  |  |  |
| PrP | 0.95(0.80,1.13) | 0.550 | 0.751 |  |  |  |  |
| BuP | 0.88(0.66,1.17) | 0.374 | 0.748 |  |  |  |  |
| HeP | 0.97(0.73,1.28) | 0.827 | 0.827 |  |  |  |  |

Binary logistic regression models were adjusted age, pre-pregnancy BMI, EPDS score, place of residence, personality, per capita monthly income, number of pregnancies, mode of conception, intended pregnancy, and presence of underlying diseases.

[Quantile g-computation](https://www.ppmy.cn/news/868180.html) was used to estimate the mixture effects of PBs. Models adjusted for age, pre-pregnancy BMI, EPDS score, place of residence, personality, per capita monthly income, number of pregnancies, mode of conception, intended pregnancy, and presence of underlying diseases.

Boldface indicates statistical significance (*P* < 0.05).

**Supplementary Table S5.** Stratified analysis of associations between prenatal paraben exposure and adverse pregnancy outcomes by maternal depressive status after excluding women with a history of more than one spontaneous abortion

|  | APOs | *P*-int | SVN | *P*-int | Macrosomia | *P*-int | Fetal distress | *P*-int | Asphyxia | *P*-int |
| --- | --- | --- | --- | --- | --- | --- | --- | --- | --- | --- |
| Depressive symptoms | | | | | | | | | | |
| Mixture | 1.07(0.76,1.51) |  | **1.51(1.00,2.26)** |  | 1.31(0.67,2.57) |  | 1.15(0.63,2.09) |  | **0.62(0.39,0.99)** |  |
| MeP | 1.08(0.87,1.35) | 0.918 | 1.23(0.96,1.58) | 0.305 | 1.06(0.69,1.64) | 0.381 | 1.11(0.80,1.54) | 0.571 | 0.77(0.54,1.09) | 0.665 |
| EtP | 1.07(0.94,1.22) | 0.920 | **1.20(1.03,1.40)** | **0.056** | 1.06(0.83,1.36) | 0.221 | 0.95(0.76,1.18) | 0.693 | 0.90(0.75,1.08) | 0.277 |
| PrP | **1.23(1.04,1.46)** | **0.045** | 1.10(0.91,1.33) | **0.086** | 1.28(0.95,1.72) | 0.620 | 1.25(0.96,1.62) | 0.348 | 0.97(0.78,1.21) | 0.420 |
| BuP | 1.12(0.85,1.48) | 0.352 | 1.31(0.96,1.77) | **0.091** | 1.13(0.71,1.80) | 0.823 | 1.13(0.75,1.72) | 0.110 | 0.73(0.48,1.10) | **0.089** |
| HeP | 0.98(0.75,1.29) | 0.900 | 1.18(0.86,1.61) | 0.107 | 1.02(0.61,1.70) | 0.691 | 0.86(0.54,1.37) | 0.302 | 0.91(0.63,1.31) | 0.451 |
| Non‑significant depressive symptoms | | | | | | | | | | |
| Mixture | 1.18(0.85,1.65) |  | 0.69(0.44,1.09) |  | **3.17(1.56,6.42)** |  | 1.18(0.67,2.07) |  | 1.23(0.62,2.41) |  |
| MeP | 1.07(0.86,1.33) |  | 0.99(0.75,1.31) |  | 1.43(0.99,2.06) |  | 0.98(0.67,1.43) |  | 0.96(0.61,1.50) |  |
| EtP | 1.08(0.95,1.23) |  | 0.95(0.80,1.14) |  | **1.33(1.03,1.71)** |  | 1.01(0.81,1.26) |  | 1.11(0.86,1.43) |  |
| PrP | 0.98(0.83,1.14) |  | 0.86(0.69,1.06) |  | **1.47(1.05,2.07)** |  | 1.03(0.79,1.34) |  | 0.90(0.66,1.22) |  |
| BuP | 0.96(0.74,1.24) |  | 0.89(0.64,1.26) |  | 1.30(0.80,2.11) |  | 0.68(0.43,1.09) |  | 1.34(0.83,2.16) |  |
| HeP | 1.04(0.81,1.34) |  | 0.75(0.52,1.07) |  | 1.35(0.82,2.22) |  | 1.21(0.80,1.82) |  | 1.19(0.74,1.91) |  |

*P*-int: *P* value for interaction. Binary logistic regression models or quantile g-computation models were adjusted for age, pre-pregnancy BMI, EPDS score, place of residence, personality, per capita monthly income, number of pregnancies, mode of conception, intended pregnancy, and presence of underlying diseases. Boldface indicates statistically significant associations (95% CI not crossing 1) or suggestive interactions (*P*-int < 0.1).

This sensitivity analysis excluded pregnant women with a history of more than one spontaneous abortion to assess the robustness of the main findings. Results are consistent with the main analysis presented in Table 3.
